# Genetic and Pharmacological Targeting of Transcriptional Repression in Resistance to Thyroid Hormone Alpha

**DOI:** 10.1089/thy.2018.0399

**Published:** 2019-05-13

**Authors:** Bernard Freudenthal, Samiksha Shetty, Natalie C. Butterfield, John G. Logan, Cho Rong Han, Xuguang Zhu, Inna Astapova, Anthony N. Hollenberg, Sheue-Yann Cheng, J.H. Duncan Bassett, Graham R. Williams

**Affiliations:** ^1^ Molecular Endocrinology Laboratory, Department of Medicine, Imperial College London, London, United Kingdom.; ^2^ Laboratory of Molecular Biology, National Cancer Institute, National Institutes of Health, Bethesda, Maryland.; ^3^ Endocrinology, Metabolism and Nutrition, Department of Medicine, Duke University School of Medicine, Durham, North Carolina.; ^4^ Joan and Sanford I. Weill Department of Medicine, Weill Cornell Medicine and New York Presbyterian/Weill Cornell Medical Center, New York, New York.

**Keywords:** thyroid hormone receptor, RTH-α, NCoR1, bone

## Abstract

***Background:*** Thyroid hormones act in bone and cartilage via thyroid hormone receptor alpha (TRα). In the absence of triiodothyronine (T3), TRα interacts with co-repressors, including nuclear receptor co-repressor-1 (NCoR1), which recruit histone deacetylases (HDACs) and mediate transcriptional repression. Dominant-negative mutations of TRα cause resistance to thyroid hormone alpha (RTHα; OMIM 614450), characterized by excessive repression of T3 target genes leading to delayed skeletal development, growth retardation, and bone dysplasia. Treatment with thyroxine has been of limited benefit, even in mildly affected individuals, and there is a need for new therapeutic strategies. It was hypothesized that (i) the skeletal manifestations of RTHα are mediated by the persistent TRα/NCoR1/HDAC repressor complex containing mutant TRα, and (ii) treatment with the HDAC inhibitor suberoylanilide hydroxamic acid (SAHA) would ameliorate these manifestations.

***Methods:*** The skeletal phenotypes of (i) *Thra1^PV/+^* mice, a well characterized model of RTHα; (ii) *Ncor1^ΔID/ΔID^* mice, which express an NCoR1 mutant that fails to interact with TRα; and (iii) *Thra1^PV/+^Ncor1^ΔID/ΔID^* double-mutant adult mice were determined. Wild-type, *Thra1^PV/+^*, *Ncor1^ΔID/ΔID^*, and *Thra1^PV/+^Ncor1^ΔID/ΔID^* double-mutant mice were also treated with SAHA to determine whether HDAC inhibition results in amelioration of skeletal abnormalities.

***Results:** Thra1^PV/+^* mice had a severe skeletal dysplasia, characterized by short stature, abnormal bone morphology, and increased bone mineral content. Despite normal bone length, *Ncor1^ΔID/ΔID^* mice displayed increased cortical bone mass, mineralization, and strength. *Thra1^PV/+^Ncor1^ΔID/ΔID^* double-mutant mice displayed only a small improvement of skeletal abnormalities compared to *Thra1^PV/+^* mice. Treatment with SAHA to inhibit histone deacetylation had no beneficial or detrimental effects on bone structure, mineralization, or strength in wild-type or mutant mice.

***Conclusions:*** These studies indicate treatment with SAHA is unlikely to improve the skeletal manifestations of RTHα. Nevertheless, the findings (i) confirm that TRα1 has a critical role in the regulation of skeletal development and adult bone mass, (ii) suggest a physiological role for alternative co-repressors that interact with TR in skeletal cells, and (iii) demonstrate a novel role for NCoR1 in the regulation of adult bone mass and strength.

## Introduction

Triiodothyronine (T3) acts mainly via thyroid hormone receptor alpha (TRα) in bone and cartilage and regulates skeletal development, postnatal growth, and the maintenance of adult bone mass, mineralization, and strength (1). Mutations affecting *THRA* encoding TRα cause resistance to thyroid hormone alpha (RTHα) (2–12), also classified as autosomal dominant non-goitrous congenital hypothyroidism type 6 (OMIM 614450).

*THRA* mutations result in a consistent pattern of thyroid function test abnormalities, comprising normal levels of thyrotropin (TSH), a low or normal thyroxine (T4), and a high or normal T3 concentration. An elevated T3/T4 ratio is pathognomonic and present in all affected individuals (10). Individuals with RTHα display a characteristic skeletal dysplasia consistent with impaired T3 action in bone and the skeletal consequences of severe congenital or juvenile hypothyroidism (1,10). These include macrocephaly with patent fontanelles and cranial sutures, delayed tooth eruption, thickened calvarium with wormian sutures, delayed ossification and bone age, epiphyseal dysgenesis, and disproportionate short stature. Affected adults have cortical hyperostosis and increased bone mineral density (BMD). A phenotype–genotype correlation has been noted in the limited number of reported cases. Missense mutations are associated with a less severe phenotype than the profound dysplasia in individuals with truncation mutations that result in expression of potent dominant-negative mutant TRα proteins (1,7,10). Consistent with this, the degree of dominant-negative activity of mutant TRα also correlates with the clinical response to treatment with thyroid hormones (6,10).

In RTHα, the mutant TRα acts as a dominant-negative repressor of T3 target gene expression and an inhibitor of wild-type TR function (2). In the absence of T3, unliganded TRα and TRβ isoforms interact with transcriptional repressors, including nuclear receptor co-repressor-1 (NCoR1). This interaction leads to recruitment of histone deacetylase (HDAC) enzymes to a co-repressor complex, resulting in chromatin remodeling and inhibition of basal T3 target gene transcription (13). Binding of T3 causes a conformational change in the receptor and disrupts the interaction between TR and NCoR1. T3 binding thus promotes recruitment of nuclear receptor co-activators, such as steroid receptor co-activator 1, which possess histone acetyl transferase activity, leading to activation of T3 target gene expression (14). In RTHα, the mutant TRα protein cannot release NCoR1 in response to T3, resulting in dominant repression of T3 target gene transcription because persistent HDAC-induced chromatin remodeling also prevents access for wild-type TRs to the transcriptional machinery. The disease phenotype in RTHα therefore reflects impaired T3 action in specific TRα-dependent target tissues such as the skeleton, and its severity is directly related to the dominant-negative potency of the mutant receptor.

It was hypothesized that (i) the skeletal manifestations of RTHα are mediated by the persistent TRα/NCoR1/HDAC repressor complex containing mutant TRα, and (ii) treatment with the HDAC inhibitor suberoylanilide hydroxamic acid (SAHA) would ameliorate the skeletal abnormalities.

To investigate these hypotheses, *Thra1^PV/+^* mice, which express a potent dominant negative mutant TR (TRα1PV) and recapitulate the RTHα phenotype observed in individuals with similar *THRA* mutations, were studied (15–17). To determine the role of NCoR1 in the pathogenesis of the skeletal manifestations of RTHα, *Thra1^PV/+^* mice were crossed with *Ncor1^ΔID/ΔID^* mice that express mutant NCoR1, which lacks the receptor interacting domains RID2 and RID3 required for TR binding but retains RID1 that interacts with other nuclear receptors (13,18,19). Finally, wild-type, *Thra1^PV/+^*, *Ncor1^ΔID/ΔID^*, and *Thra1^PV/+^Ncor1^ΔID/ΔID^* double-mutant mice were treated with SAHA to determine whether HDAC inhibition results in amelioration of skeletal abnormalities in RTHα.

## Methods

### Animals and treatment

Animal studies were performed according to protocols approved by the National Cancer Institute Animal Care and Use Committee. Heterozygous *Thra1^PV/+^* mice were generated in a mixed C57BL/6J and NIH Black Swiss genetic background and genotyped as described (17). Homozygous *Ncor1^ΔID/ΔID^* mice were generated in a mixed C57BL/6 and 129S6 background as described (20). *Thra1^PV/+^* mice and *Ncor1^ΔID/ΔID^* mice were inter-crossed for several generations to produce *Thra1^PV/+^Ncor1^ΔID/ΔID^* double mutants in a mixed C57BL/6J, NIH Black Swiss and 129S6 genetic background (21). Wild-type mice in a mixed C57BL/6J, NIH Black Swiss and 129S6 genetic background were used to ensure comparisons were made in as similar a genetic background as possible for all experiments (*n* = 4–8 per group).

SAHA (Selleck Chemicals, Houston, TX) or vehicle was prepared as described (22). A daily dose of 50 mg/kg body weight was administered by oral gavage for a two-month period starting at the age of six weeks until bones were harvested at 14 weeks of age.

### Faxitron digital X-ray microradiography

Dissected femurs, humeri, and proximal caudal vertebrae Ca6 and Ca7 were imaged at 10 μm resolution using a Faxitron MX20 (Qados; Cross Technologies plc, Sandhurst, United Kingdom). Bone lengths were determined after calibrating images with a digital micrometer using ImageJ (https://imagej.nih.gov/ij/). Bone mineral content (BMC) was determined relative to steel, aluminum, and polyester standards, and images were pseudocolored using a 16-color lookup table (23,24).

### Micro computed tomography

Femurs were imaged by micro computed tomography using a SCANCO μCT 50 (SCANCO Medical AG, Bruttisellen, Switzerland) at 70 kV, 200 μA, with a 0.5 mm aluminum filter and voxel resolutions of 5 and 10 μm for trabecular and cortical bone, respectively. Images were reconstructed and analyzed using Scanco software. A 1 mm^3^ region of interest was selected 100 μm from the growth plate, and trabecular bone volume as proportion of tissue volume (BV/TV), trabecular number (Tb.N), trabecular thickness (Tb.Th), and trabecular spacing (Tb.Sp) were determined (23,25,26). Total cross-sectional area (Tt.Ar), cortical bone area (Ct.Ar), marrow or medullary area (Ma.Ar), and cortical area fraction (Ct.Ar/Tt.Ar) were also determined. A 1.5 mm region of interest, centered in the midshaft 56% along the length of the femur distal to the femoral head, was selected to determine cortical thickness (Ct.Th) and cortical BMD. Cortical porosity (Ct.Po), periosteal perimeter (Ps.Pm), and endocortical perimeter (Ec.Pm) were determined within a 0.5 mm region of interest centered in the midshaft 56% along the length of the femur distal to the femoral head from images with a voxel resolution of 1 μm.

### Biomechanical testing

Destructive three-point bend tests were performed on 70% ethanol-fixed humeri to determine bone strength using an Instron 5543 load frame and 100 N load cell (Instron Limited, High Wycombe, United Kingdom). Humeri were positioned horizontally on custom supports, and load was applied perpendicular to the mid-diaphysis at a constant rate of displacement of 0.03 mm/s until fracture. Yield load, maximum load, fracture load, and stiffness were determined from load displacement curves (23,27).

### Statistical analysis

Data were normally distributed and analyzed by analysis of variance and Tukey's *post hoc* test or unpaired two-tailed Student's *t*-test. *p*-Values <0.05 were considered statistically significant. Cumulative and relative frequency distributions of BMC were compared using the Kolmogorov–Smirnov test (23,24).

## Results

### Expression of NCoR1ΔID ameliorates bone structural defects in *Thra1^PV/+^* mice and increases bone strength

**FIG. 1.**
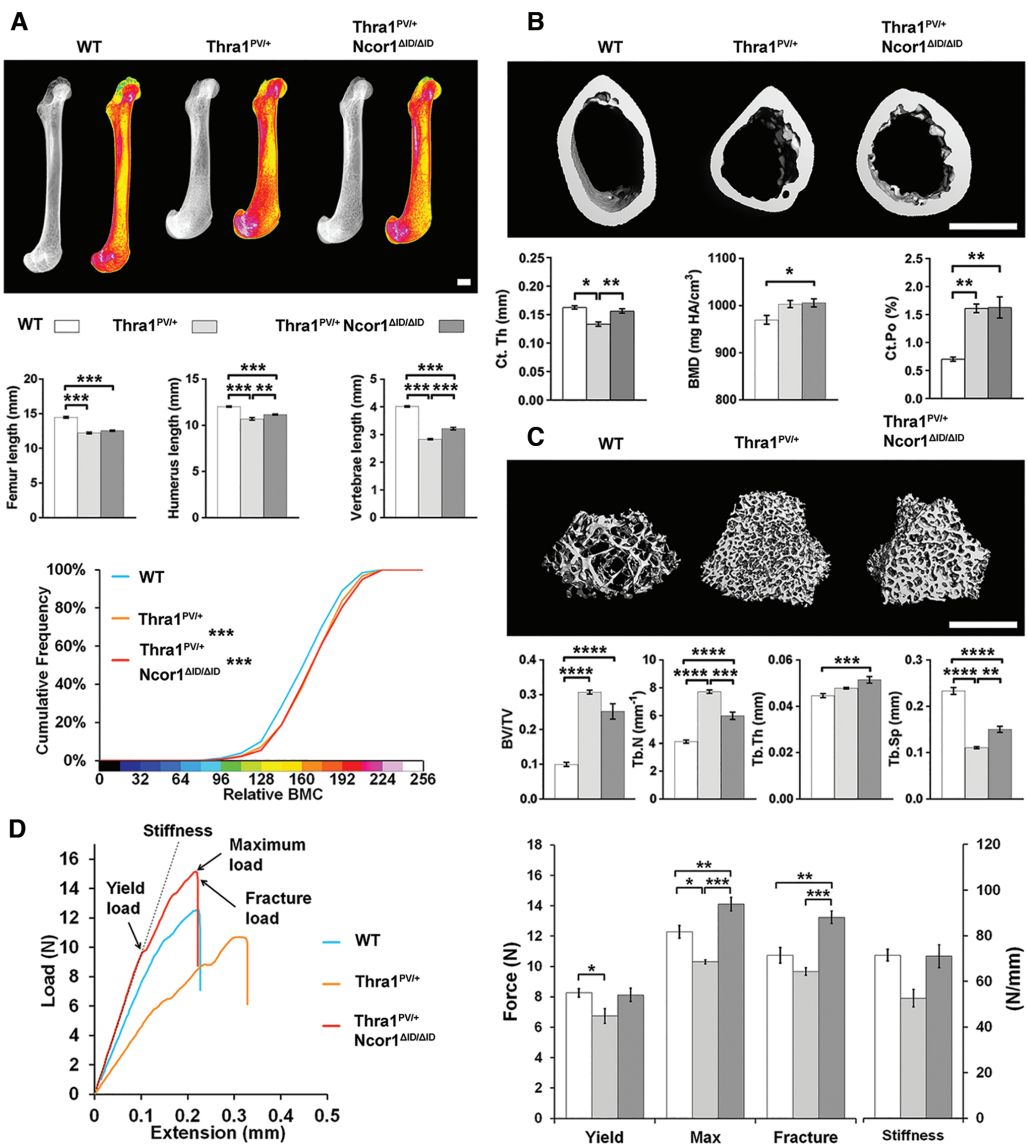
Disruption of the interaction between nuclear receptor co-repressor-1 (NcoR1) and thyroid hormone receptor alpha (TRα) ameliorates the skeletal phenotype in *Thra1^PV/+^* mice. (**A**) X-ray microradiography images of femurs from male wild-type (WT), *Thra1^PV/+^*, and *Thra1^PV/+^NCoR1^ΔID/ΔID^* mice at 14 weeks of age. Gray-scale images and pseudocolored images in which low bone mineral content (BMC) is indicated in green and high BMC in pink. Cumulative frequency histogram of relative BMC (*n* = 5–8 per group). ***p < 0.001 vs. WT; Kolmogorov–Smirnov test. Graphs show lengths of femurs, humeri, and vertebrae. Data are shown as the mean ± standard error of the mean (SEM; *n* = 4–8 per group). **p < 0.01 and ***p < 0.001; analysis of variance (ANOVA) followed by Tukey's *post hoc* test. (**B**) Micro computed tomography (micro-CT) images of mid-diaphysis cortical bone. Graphs showing cortical thickness (Ct.Th), cortical bone mineral density (BMD), and cortical porosity (Ct.Po). Data are shown as the mean ± SEM (*n* = 5–8 per group apart from Ct.Po, *n* = 3). *p < 0.05 and **p < 0.01; ANOVA followed by Tukey's *post hoc* test. (**C**) Micro-CT images of distal femur trabecular bone. Graphs showing trabecular bone volume/tissue volume (BV/TV), trabecular number (Tb.N), trabecular thickness (Tb.Th), and trabecular spacing (Tb.Sp). Data are shown as the mean ± SEM (*n* = 5–8 per group). **p < 0.01, ***p < 0.001, and ****p < 0.0001; ANOVA followed by Tukey's *post hoc* test. (**D**) Representative load displacement curves for humerus three-point bend testing. Yield load, maximum load, fracture load, and stiffness. Data are the mean ± SEM (*n* = 5–8 per group). *p < 0.05, **p < 0.01, and ***p < 0.001; ANOVA followed by Tukey's *post hoc* test. Scale bars in (**A**), (**B**) and (**C**) = 1 mm. Data from the same group of untreated WT mice are included in Figures 1–4 to facilitate comparison across groups. Color images are available online.

In 14-week-old adult *Thra1^PV/+^* mice, the lengths of the femurs, humeri, and vertebrae were decreased by 12%, 16%, and 30%, respectively, and BMC was increased (*p* < 0.001). The changes in bone length and BMC were accompanied by morphological abnormalities that included dysmorphic epiphyses with misshapen joints and splayed metaphyses with defective inwasting (Fig. 1A). Similar dysplastic features were evident in *Thra1^PV/+^Ncor1^ΔID/ΔID^* double-mutant mice, in which bone lengths were also decreased and BMC increased compared to wild-type mice. Nevertheless, reductions in the lengths of the humerus and vertebrae in *Thra1^PV/+^Ncor1^ΔID/ΔID^* double mutants were less than those observed in *Thra1^PV/+^* mice (Fig. 1A). Femurs from *Thra1^PV/+^* mice had reduced Ct.Th and increased Ct.Po but no difference in BMD compared to wild-type mice (Fig. 1B). These parameters were accompanied by increased Tt.Ar, Ma.Ar, Ps.Pm, and Ec.Pm, no difference in Ct.Ar, and a decrease in Ct.Ar/Tt.Ar (Supplementary Fig. S1), indicating an overall decrease in cortical bone thickness together with increased porosity but normal BMD. *Thra1^PV/+^* mice also had high trabecular bone mass, as evidenced by a threefold increase in BV/TV, a twofold increase in Tb.N, and a twofold reduction in Tb.Sp (Fig. 1C). *Thra1^PV/+^Ncor1^ΔID/ΔID^* double-mutant mice had a small increase in BMD and increased Ct.Po (Fig. 1B) but high trabecular bone mass, with a 2.5-fold increase in BV/TV, a 50% increase in Tb.N, a 10% increase in Tb.Th, and a 62.5% reduction in Tb.Sp (Fig. 1C). The changes in cortical and trabecular bone parameters were not as large in *Thra1^PV/+^Ncor1^ΔID/ΔID^* double mutants compared to *Thra1^PV/+^* mice (Fig. 1B and C), other than an increase in cortical area (Supplementary Fig. S1). The structural abnormalities in *Thra1^PV/+^* mice resulted in humeri that were weak, with an 18% decrease in yield load and 16% decrease in maximum load, whereas humeri from *Thra1^PV/+^Ncor1^ΔID/ΔID^* double-mutant mice were stronger, with a 16% increase in maximum load and 24% increase in fracture load compared to wild-type mice (Fig. 1D).

Overall, expression of NCoR1ΔID in double-mutant mice ameliorated the structural abnormalities evident in bones from *Thra1^PV/+^* mice and ultimately resulted in increased adult bone strength, consistent with the increased cortical area observed in *Thra1^PV/+^Ncor1^ΔID/ΔID^* double mutants.

### Expression of NCoR1ΔID increases bone mass, mineralization, and strength

**FIG. 2.**
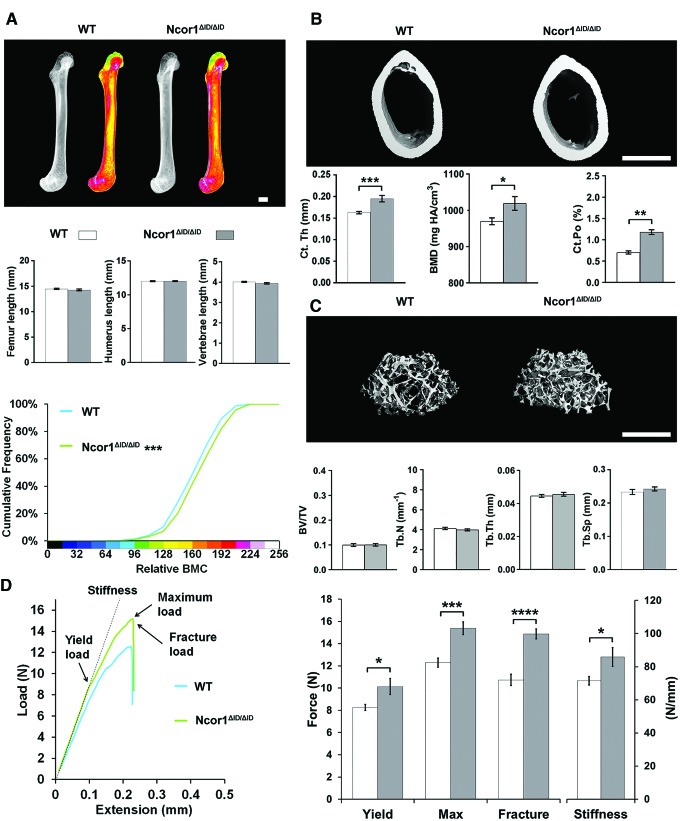
Disruption of the interaction between NcoR1 and TRα increases bone mass, mineralization, and strength in WT mice. (**A**) X-ray microradiography images of femurs from male WT and *NCoR1^ΔID/ΔID^* mice at 14 weeks of age. Gray-scale images and pseudocolored images in which low BMC is indicated in green and high BMC in pink. Cumulative frequency histogram of relative BMC (*n* = 6–8 per group). ***p < 0.001 vs. WT; Kolmogorov–Smirnov test. Graphs show lengths of femurs, humeri, and vertebrae. Data are shown as the mean ± SEM (*n* = 6–8 per group); Student's *t*-test. (**B**) Micro-CT images of mid-diaphysis cortical bone. Graphs showing Ct.Th, cortical BMD, and Ct.Po. Data are shown as the mean ± SEM (*n* = 6–8 per group apart from Ct.Po, *n* = 3). *p < 0.05, **p < 0.01, and ***p < 0.001; Student's *t*-test. (**C**) Micro-CT images of distal femur trabecular bone. Graphs showing BV/TV, Tb.N, Tb.Th, and Tb.Sp. Data are shown as the mean ± SEM (*n* = 6–8 per group). (**D**) Representative load displacement curves for humerus three-point bend testing. Yield load, maximum load, fracture load, and stiffness. Data are the mean ± SEM (*n* = 6–8 per group). *p < 0.05, ***p < 0.001, and ****p < 0.0001 vs. WT; Student's *t*-test. Scale bars in (**A**), (**B**) and (**C**) = 1 mm. Data from the same group of untreated WT mice are included in Figures 1–4 to facilitate comparison across groups. Color images are available online.

In 14-week-old adult *Ncor1^ΔID/ΔID^* mice, skeletal morphology and the lengths of the femurs, humeri, and vertebrae were similar to wild-type mice, but femurs had increased BMC (Fig. 2A) that resulted from a combination of an 18% increase in Ct.Th, a small increase in cortical BMD, and an increase in Ct.Po (Fig. 2B). There were no differences in trabecular bone parameters (Fig. 2C). The increased cortical bone in *Ncor1^ΔID/ΔID^* mice (Fig. 2B and Supplementary Fig. S2) resulted in increased bone strength compared to wild-type mice, as evidenced by a 30% increase in maximum load, a 35% increased fracture load, and a 20% increase in stiffness (Fig. 2D).

Overall, expression of NCoR1ΔID results in increased cortical bone mass, mineralization, and strength in adult *Ncor1^ΔID/ΔID^* mice despite an increase in Ct.Po.

### Treatment with SAHA has no effect on bone mass, mineralization, or strength

**FIG. 3.**
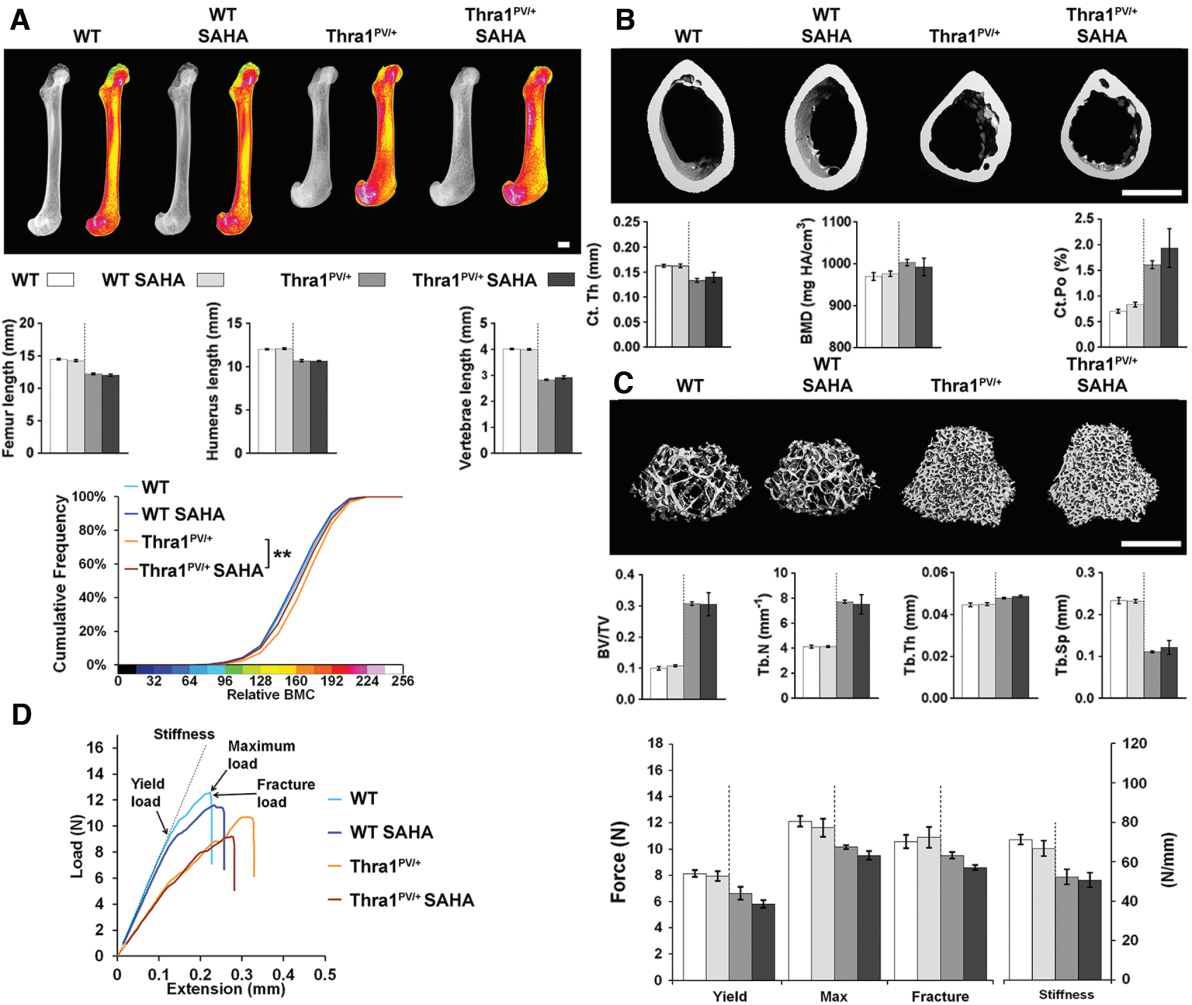
Treatment with suberoylanilide hydroxamic acid (SAHA) has no effect on bone mass, mineralization, or strength in WT or *Thra1^PV/+^* mice. (**A**) X-ray microradiography images of femurs from male WT, SAHA-treated WT (WT SAHA), *Thra1^PV/+^*, and SAHA-treated *Thra1^PV/+^* (*Thra1^PV/+^* SAHA) mice at 14 weeks of age. Gray-scale images and pseudocolored images in which low BMC is indicated in green and high BMC in pink. Cumulative frequency histogram of relative BMC (*n* = 5–8 per group). **p < 0.01 SAHA treated vs. untreated; Kolmogorov–Smirnov test. Graphs show lengths of femurs, humeri, and vertebrae. Data are shown as the mean ± SEM (*n* = 5–8 per group), treated vs. untreated; Student's *t*-test. (**B**) Micro-CT images of mid-diaphysis cortical bone. Graphs showing Ct.Th, cortical BMD, and Ct.Po. Data are shown as the mean ± SEM (*n* = 5–8 per group apart from Ct.Po, *n* = 3). (**C**) Micro-CT images of distal femur trabecular bone. Graphs showing BV/TV, Tb.N, Tb.Th, and Tb.Sp. Data are shown as the mean ± SEM (*n* = 5–8 per group). (**D**) Representative load displacement curves for humerus three-point bend testing. Yield load, maximum load, fracture load, and stiffness. Data are the mean ± standard error of the mean (*n* = 5–8 per group); treated vs. untreated. Scale bars in (**A**), (**B**) and (**C**) = 1 mm. Data from the same group of untreated WT mice are included in Figures 1–4 to facilitate comparison across groups. Color images are available online.

**FIG. 4.**
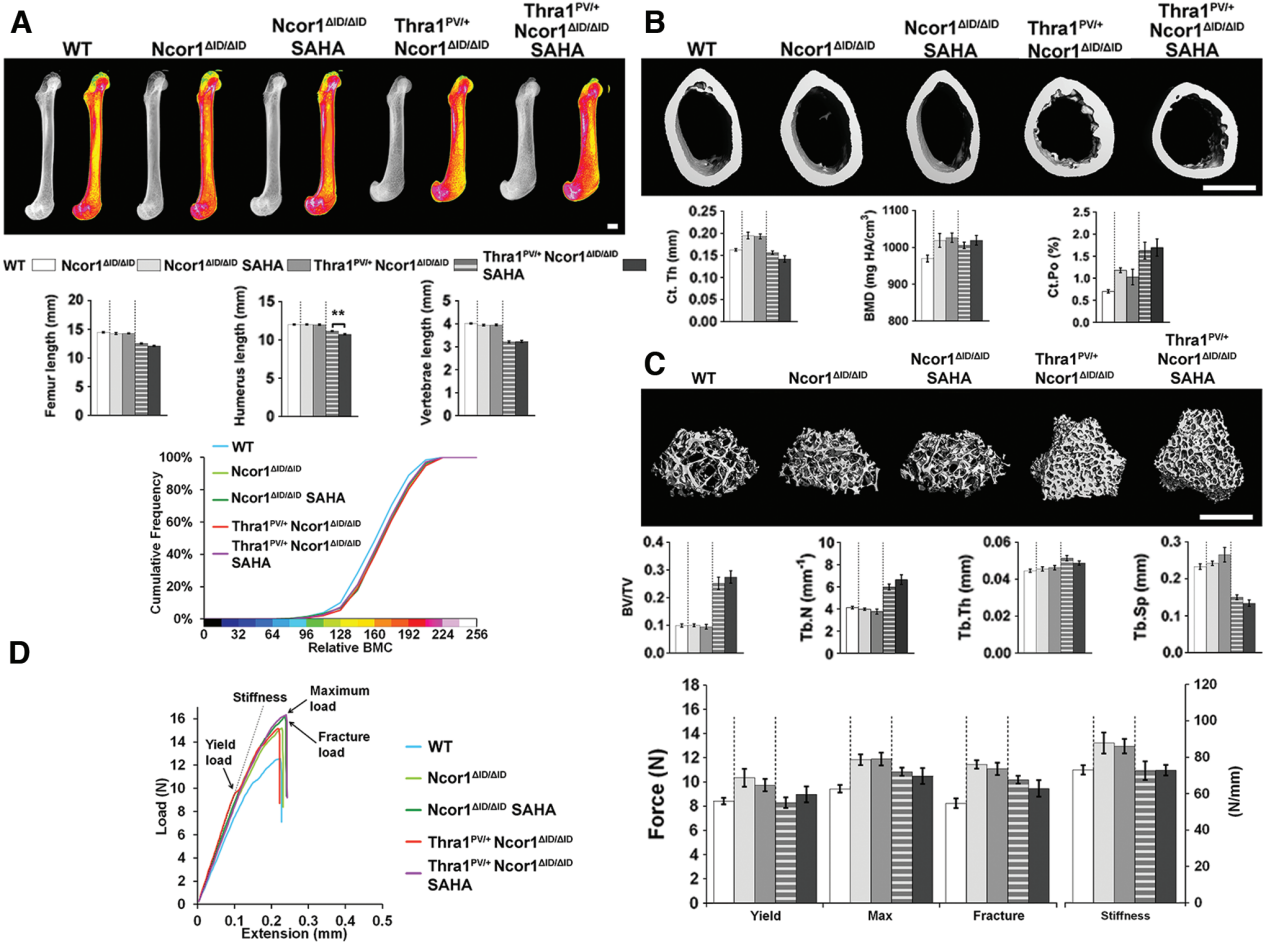
Treatment with SAHA has no effect on bone mass, mineralization, or strength in *NCoR1^ΔID/ΔID^* or *Thra1^PV/+^NCoR1^ΔID/ΔID^* mice. (**A**) X-ray microradiography images of femurs from male WT, *NCoR1^ΔID/ΔID^*, SAHA-treated *NCoR1^ΔID/ΔID^* (*NCoR1^ΔID/ΔID^* SAHA), *Thra1^PV/+^NCoR1^ΔID/ΔID^*, and SAHA-treated *Thra1^PV/+^NCoR1^ΔID/ΔID^* (*Thra1^PV/+^NCoR1^ΔID/ΔID^* SAHA) mice at 14 weeks of age. Gray-scale images and pseudocolored images in which low BMC is indicated in green and high BMC in pink. Cumulative frequency histogram of relative BMC (*n* = 6–8 per group). SAHA treated vs. untreated; Kolmogorov–Smirnov test. Graphs show femur, humerus and vertebral lengths. Data are shown as the mean ± SEM (*n* = 6–8 per group); treated vs. untreated. **p < 0.01; Student's *t*-test. (**B**) Micro-CT images of mid-diaphysis cortical bone. Graphs showing Ct.Th, cortical BMD, and Ct.Po. Data are shown as the mean ± SEM (*n* = 6–8 per group apart from Ct.Po, *n* = 3). (**C**) Micro-CT images of distal femur trabecular bone. Graphs showing BV/TV, Tb.N, Tb.Th, and Tb.Sp. Data are shown as the mean ± SEM (*n* = 6–8 per group). (**D**) Representative load displacement curves for humerus three-point bend testing. Yield load, maximum load, fracture load, and stiffness. Data are the mean ± SEM (*n* = 6–8 per group); treated vs. untreated. Scale bars in (**A**), (**B**) and (**C**) = 1 mm. Data from the same group of untreated WT mice are included in Figures 1–4 to facilitate comparison across groups. Color images are available online.

Two months of treatment with the histone deacetylase inhibitor SAHA between 6 and 14 weeks of age had no effect on bone structure, mineralization, or strength in either wild-type or *Thra1^PV/+^* mice, other than a small decrease in femur BMC in *Thra1^PV/+^* mice (Fig. 3 and Supplementary Fig. S3). Similarly, treatment of *Ncor1^ΔID/ΔID^* and *Thra1^PV/+^Ncor1^ΔID/ΔID^* double-mutant mice with SAHA had no effect on any parameter, other than a small reduction in the length of the humerus present in *Thra1^PV/+^Ncor1^ΔID/ΔID^* double-mutant mice treated with SAHA (Fig. 4 and Supplementary Fig. S4).

Overall, treatment with SAHA had no beneficial or detrimental effect on bone structure, mineralization, or strength in wild-type or mutant mice.

## Discussion

RTHα results in delayed skeletal and dental development, growth retardation, and skeletal dysplasia, as well as additional non-skeletal manifestations primarily affecting the central nervous, gastro-intestinal, and metabolic systems (10). To date, 11 individuals have received treatment with T4, but other therapeutic possibilities have not been investigated (2–5,8,9,12). Children with less severe frameshift *THRA* mutations have derived only limited benefit after treatment with T4, particularly with respect to growth (2,8,12), but no skeletal responses have been seen in individuals with deleterious frame-shift truncation mutations (6,9,28). Thus, there is a need to investigate new therapeutic strategies for treatment of the debilitating skeletal manifestations of RTHα. Here, two complementary approaches to disrupt the detrimental dominant-negative activity of mutant TRα in the *Thra1^PV/+^* mouse model of RTHα were investigated.

First, T3 action in bone is mediated by the canonical genomic actions of TRα1 (1,26), while the skeletal dysplasia in RTHα results from (i) impaired tissue T3 responsiveness in bone and cartilage despite a moderately increased circulating T3 concentration and (ii) the dominant-negative repressor activity of mutant TRα1 that results from its inability to release co-repressor in the presence of T3 (13). Normally, NCoR1 interacts with TR via two interaction domains and is the main co-repressor that recruits HDACs to mediate transcriptional repression in the absence of T3. The functional interaction between TRα1 and NCoR1 can be targeted elegantly in mice by the *Ncor1^ΔID^* mutation (13,18,19), which prevents association between NCoR1 and unliganded TR and thus relieves NCoR1-dependent transcriptional repression (21,29–31).

It was previously shown that similar to patients with RTHα, *Thra1^PV/+^* mice have an abnormal hypothalamic–pituitary–thyroid (HPT) axis, with a moderately increased T3 concentration, normal T4, slightly elevated TSH, and an increased size of the thyroid gland (21). *Ncor1^ΔID/ΔID^* mice by contrast have decreased T3 and T4 levels but a normal TSH and normal sized thyroid gland. *Thra1^PV/+^Ncor1^ΔID/ΔID^* double-mutant mice have a normal T3, decreased T4, normal TSH, and a slightly enlarged thyroid gland. Thus, co-expression of NCoR1ΔID in *Thra1^PV/+^Ncor1^ΔID/ΔID^* double mutants ameliorates dysregulation of the HPT axis and normalizes the increased T3 and TSH levels that are caused by dominant-negative actions of TRα1PV in *Thra1^PV/+^* mice (21). By contrast, treatment of *Thra1^PV/+^*, *Ncor1^ΔID/ΔID^*, and *Thra1^PV/+^Ncor1^ΔID/ΔID^* double-mutant mice with SAHA does not affect circulating T3, T4, or TSH concentrations (22).

Disruption of the interaction between TRα1 and NCoR1 in *Ncor1^ΔID/ΔID^* mice resulted in increased cortical bone mass, mineralization, and strength but did not affect linear growth or trabecular bone parameters (Fig. 2). These findings demonstrate a new and important physiological and homeostatic function of NCoR1 affecting the skeleton. Thus, a lack of interaction between NCoR1 and TR isoforms has an anabolic impact on cortical bone to improve bone strength, whereas global interactions between NCoR1 and TRs limit cortical bone accumulation and decrease bone strength. They also reveal that NCoR1 is dispensable during endochondral bone formation and postnatal growth. Consistent with this new role for NCoR1 in bone, disruption of the interaction between dominant-negative TRα1^PV^ and NCoR1 in *Thra1^PV/+^Ncor1^ΔID/ΔID^* double-mutant mice also increased cortical bone mass and strength but had only limited effects on skeletal morphology, linear growth, and trabecular bone mass (Fig. 1). Importantly, comparison of *Ncor1^ΔID/ΔID^* mice (Fig. 2) with *Thra1^PV/+^Ncor1^ΔID/ΔID^* double-mutant mice (Fig. 1) reveals that *Thra1^PV/+^Ncor1^ΔID/ΔID^* mice have decreased bone strength parameters. This effect of TRα1^PV^ to worsen the phenotype of *Ncor1^ΔID/ΔID^* mice is independent of NCoR1 function and thus provides evidence of a role for additional co-repressors that may interact with TRα in skeletal cells.

Our second approach was to investigate pharmacological inhibition of excessive HDAC activity in RTHα. SAHA chelates zinc ions required for histone deacetylase activity and transcriptional repression, thus resulting in enzyme inhibition, increased histone acetylation, and transcriptional activation (32). SAHA has received approval from the U.S. Food and Drug Administration for the treatment of certain types of cancer and thus could be repurposed for other disease applications if effective. Accordingly, SAHA has been proposed as a potential drug to relieve the detrimental consequences of transcriptional repression in RTHα, and has been shown to ameliorate impaired adipogenesis in *Thra1^PV/+^* mice at the same dose used in the current study (22). Despite this, it was found that pharmacological inhibition of excessive HDAC activity in *Thra1^PV/+^* and *Thra1^PV/+^Ncor1^ΔID/ΔID^* double-mutant mice had no beneficial effect on linear growth or adult bone structure and strength (Figs. 3 and 4). This lack of skeletal response to SAHA may be due to (i) its short half-life following once-daily oral administration in mice (33), (ii) commencement of treatment at six weeks of age after the period of maximum linear growth velocity (34), and (iii) its potentially limited bioavailability in bone and cartilage. Overall, the current data suggest that SAHA is unlikely to have therapeutic benefit in the treatment of skeletal manifestations in RTHα.

In summary, the current studies demonstrate that TRα1 exerts major regulatory effects on linear growth and adult bone turnover that are independent of NCoR1. Overall, the findings suggest that the skeletal manifestations of RTHα are not mediated by persistent interactions between mutant TRα with NCoR1 and HDAC. The limited improvement of skeletal abnormalities in RTHα following disruption of the interaction between the dominant-negative TR and NCoR1 further suggests an important role for alternative co-repressors that interact with TR in skeletal cells such as silencing mediator of retinoid and thyroid hormone receptors (13).

Despite this, these studies identify a novel physiological role for NCoR1 in association with TRs to (i) optimize bone strength and (ii) limit excessive accumulation of cortical bone.

## Supplementary Material

Supplemental data

Supplemental data

Supplemental data

Supplemental data
