# Supplementary material for: Genetic and Pharmacological Targeting of Transcriptional Repression in Resistance to Thyroid Hormone Alpha
Source: Thyroid. 2019 May 13;29(5):726–34. doi: 10.1089/thy.2018.0399 (PMC6533791; doi:10.1089/thy.2018.0399)
Supplement: Supplemental data [file Supp_Fig1.pdf]

Supplementary Data

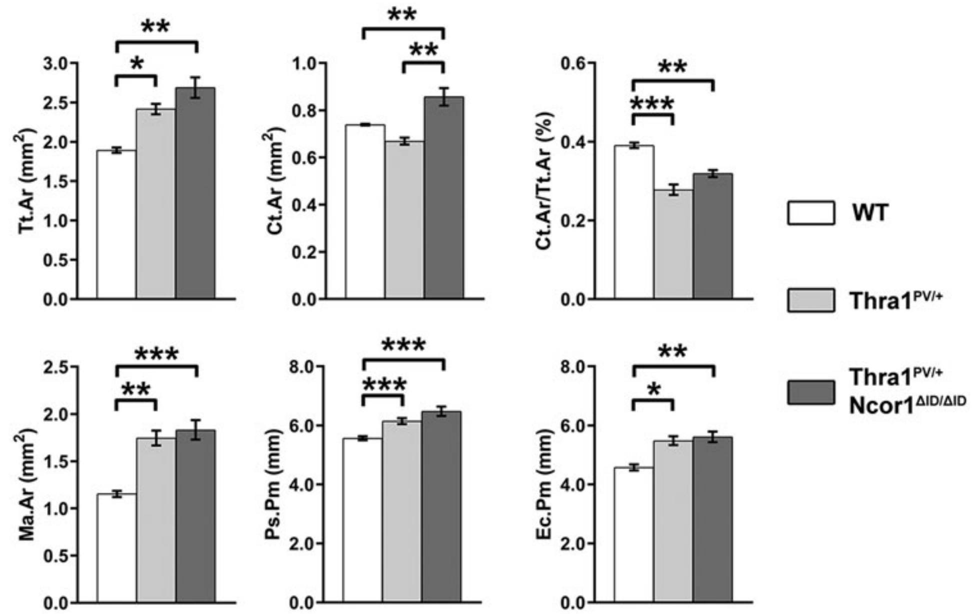

SUPPLEMENTARY FIG. S1. Disruption of the interaction between NcoR1 and TRα ameliorates the skeletal phenotype in *Thra1*$^{PV/+}$ mice—additional cortical micro-CT analyses. Graphs showing total cross-sectional area inside the periosteal envelope (Tt.Ar), cortical bone area (Ct.Ar), cortical area fraction (Ct.Ar/Tt.Ar), medullary (or marrow) area (Ma.Ar), periosteal perimeter (Ps.Pm), and endocortical perimeter (Ec.Pm) from male wild-type (WT), *Thra1*$^{PV/+}$, and *Thra1*$^{PV/+}$*NCoR1*$^{\Delta ID/\Delta ID}$ mice at 14 weeks of age ($n=3$ per genotype). Data are shown as the mean ± standard error of the mean (SEM). *$p<0.05$, **$p<0.01$, and ***$p<0.001$; ANOVA followed by Tukey's *post hoc* test.
