# Supplementary material for: Genetic and Pharmacological Targeting of Transcriptional Repression in Resistance to Thyroid Hormone Alpha
Source: Thyroid. 2019 May 13;29(5):726–34. doi: 10.1089/thy.2018.0399 (PMC6533791; doi:10.1089/thy.2018.0399)
Supplement: Supplemental data [file Supp_Fig2.pdf]

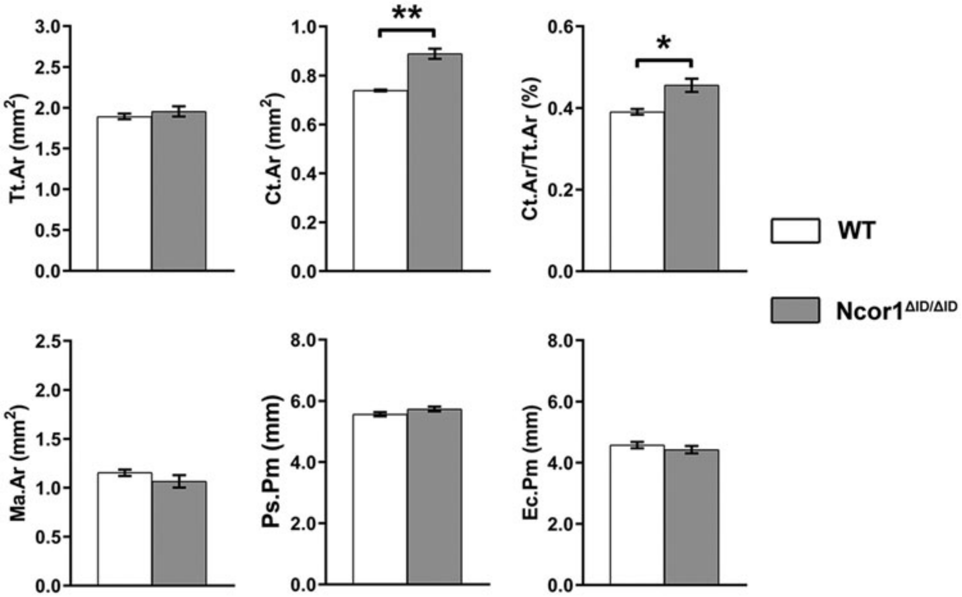

SUPPLEMENTARY FIG. S2. Disruption of the interaction between NcoR1 and TRα increases bone mass, mineralization, and strength in WT mice—additional cortical micro-CT analyses. Graphs showing Tt.Ar, Ct.Ar, Ct.Ar/Tt.Ar, Ma.Ar, Ps.Pm, and Ec.Pm from male WT and $NCoR1^{\Delta ID/\Delta ID}$ mice at 14 weeks of age ($n = 3$ per genotype). Data are shown as the mean ± SEM. $^{*}p < 0.05$ and $^{**}p < 0.01$; Student's *t*-test.
