# Supplementary material for: Genetic and Pharmacological Targeting of Transcriptional Repression in Resistance to Thyroid Hormone Alpha
Source: Thyroid. 2019 May 13;29(5):726–34. doi: 10.1089/thy.2018.0399 (PMC6533791; doi:10.1089/thy.2018.0399)
Supplement: Supplemental data [file Supp_Fig3.pdf]

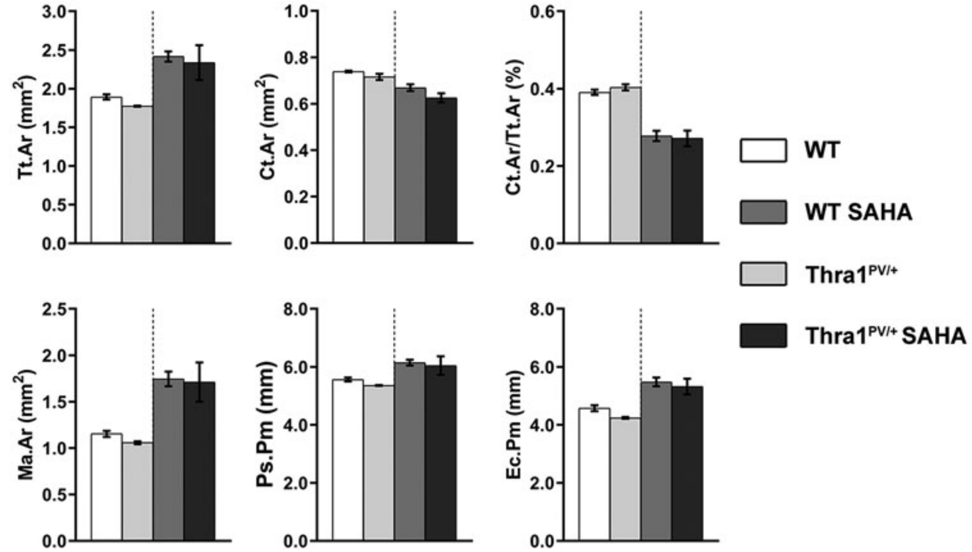

SUPPLEMENTARY FIG. S3. Treatment with SAHA has no effect on bone mass, mineralization, or strength in WT or *Thra1*$^{PV/+}$ mice—additional cortical micro-CT analyses. Graphs showing Tt.Ar, Ct.Ar, Ct.Ar/Tt.Ar, Ma.Ar, Ps.Pm, and Ec.Pm from male WT, SAHA-treated WT (WT SAHA), *Thra1*$^{PV/+}$, and SAHA-treated *Thra1*$^{PV/+}$ (*Thra1*$^{PV/+}$ SAHA) mice at 14 weeks of age ($n=3$ per genotype). Data are shown as the mean ± SEM.
