# Supplementary material for: Genetic and Pharmacological Targeting of Transcriptional Repression in Resistance to Thyroid Hormone Alpha
Source: Thyroid. 2019 May 13;29(5):726–34. doi: 10.1089/thy.2018.0399 (PMC6533791; doi:10.1089/thy.2018.0399)
Supplement: Supplemental data [file Supp_Fig4.pdf]

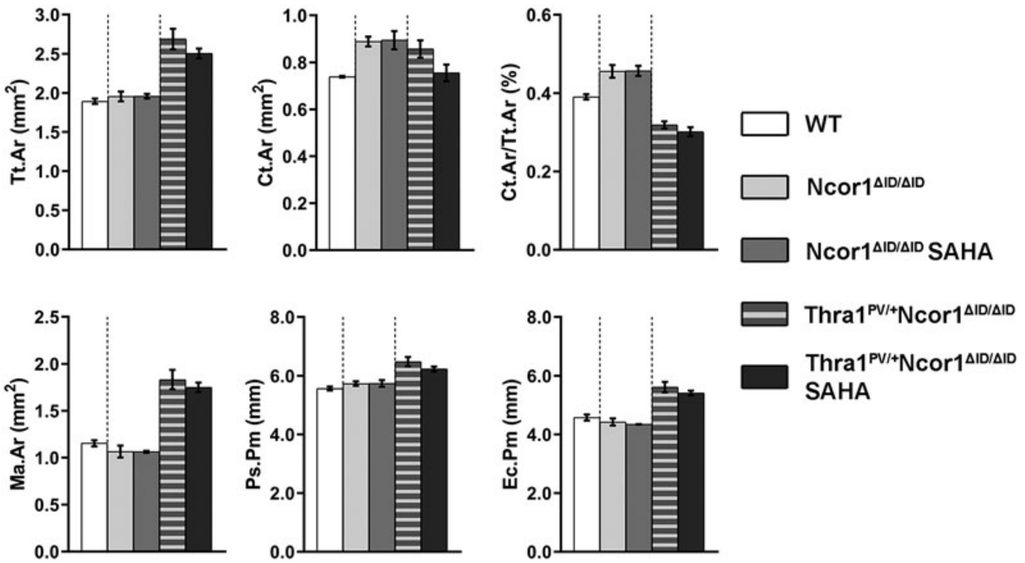

SUPPLEMENTARY FIG. S4. Treatment with SAHA has no effect on bone mass, mineralization, or strength in *NCoR1*$^{\Delta ID/\Delta ID}$ or *Thra1*$^{PV/+}$*NCoR1*$^{\Delta ID/\Delta ID}$ mice—additional cortical micro-CT analyses. Graphs showing Tt.Ar, Ct.Ar, Ct.Ar/Tt.Ar, Ma.Ar, Ps.Pm, and Ec.Pm from male WT, *NCoR1*$^{\Delta ID/\Delta ID}$, SAHA-treated *NCoR1*$^{\Delta ID/\Delta ID}$ (*NCoR1*$^{\Delta ID/\Delta ID}$ SAHA), *Thra1*$^{PV/+}$*NCoR1*$^{\Delta ID/\Delta ID}$, and SAHA-treated *Thra1*$^{PV/+}$*NCoR1*$^{\Delta ID/\Delta ID}$ (*Thra1*$^{PV/+}$*NCoR1*$^{\Delta ID/\Delta ID}$ SAHA) mice at 14 weeks of age ($n = 3$ per genotype). Data are shown as the mean ± SEM.
